# Unravelling physiological disorders in onion and garlic: critical assessment and bibliometric visualization

**DOI:** 10.3389/fpls.2024.1500917

**Published:** 2024-12-13

**Authors:** Rajiv B. Kale, Kiran Khandagale, Sendhil Ramadas, Abhishek Dilip Gavhane, Pranjali Gedam, Vijay Mahajan

**Affiliations:** ^1^ ICAR-Directorate of Onion and Garlic Research, Pune, India; ^2^ Department of Economics, Pondicherry University, Puducherry, India

**Keywords:** *Allium cepa*, *Allium sativum*, bibliometrics, bolting, sprouting, watery scale, doubles, rubberization

## Abstract

Onion and garlic are economically important vegetable crops cultivated worldwide. Numerous pests and diseases affect the quality and yield of these crops. In addition to diseases and pests, several physiological disorders affect onion and garlic. The physiological disorders are abnormalities caused by intercultural operations, nutrient management, environmental factors, genetic regulation, etc. These physiological disorders significantly affect the yield and quality of onion and garlic, leading to monetary losses to the farmers. The following physiological disorders are commonly reported in onion: premature bolting, sprouting in storage, watery scale, doubling/twins, basal plate split, and thick neck. Premature sprouting and rubberization are the main physiological anomalies observed in garlic. The present review described the symptoms of these physiological abnormalities, the factors responsible, and ways to avoid or minimise the occurrence of these abnormalities to subsequently reduce the losses of the growers. Further, we also performed bibliometric analysis using the SCOPUS database. This is the first review that describes the progress of research on physiological disorders in onion and garlic in detail, which will positively increase awareness about such important aspects of onion and garlic. Further, it will provide insight to researchers for developing innovative strategies, cultural practices, and varieties to control these physiological abnormalities of onion and garlic.

## 1 Introduction

Onion (*Allium cepa*) and garlic (*Allium sativum*) are important vegetables, condiment and spice crops. The onion and garlic stand as one of the most ancient vegetables, and their presence is documented in numerous ancient scriptures (Basak, 1987; Kahn, 1996; Khandagale and Gawande 2019). By the time of the Middle Ages, it had cemented its place as a culinary staple in many cuisines worldwide, ensuring a year-round demand. Remarkably, the onion ranks as the third most crucial horticultural crop, trailing only behind the potato and tomato, and is cultivated commercially in over 170 countries across the globe (Teshika et al., 2018). The global onion production was 107 million tonnes in 2021 from 5.78 million hectares area. India is a leader in onion production, with 31.6 million tonnes harvested from an area of 19.41 hundred thousand hectares (Kale et al., 2024). Garlic, a ubiquitous *Allium* species with a long culinary history, is a globally significant crop according to the Food and Agriculture Organisation (FAOSTAT, 2021). Global production reached 28 million metric tonnes from 16.36 million hectares, with China dominating the landscape. China cultivated over half the global garlic area (8.3 million hectares), producing a staggering 207.57 million tonnes. Despite China’s dominance, other players like India (29.17 million tonnes) contribute a significant share of global production.

**Figure 1:**
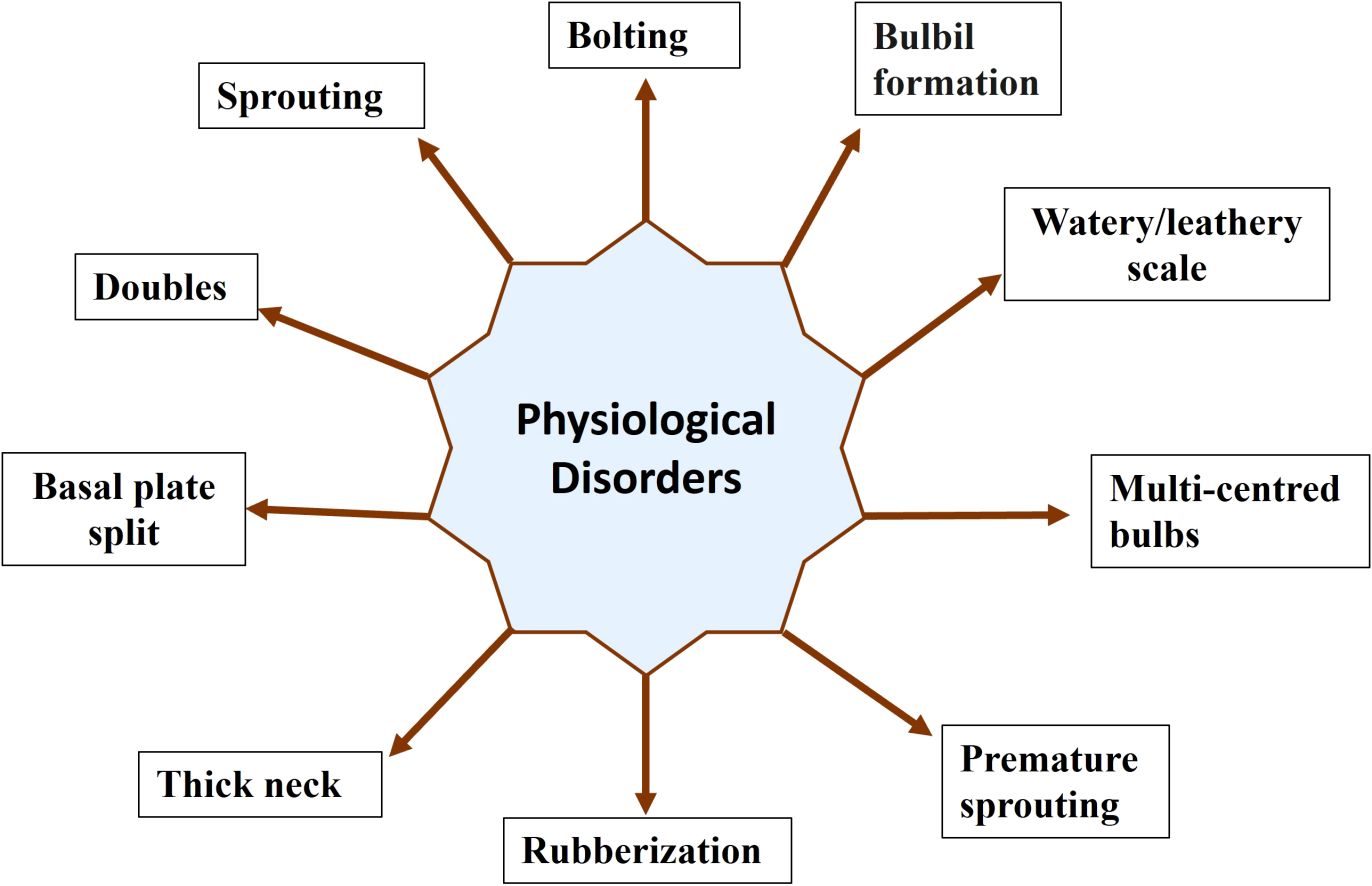
Different physiological disorders in onion and garlic.

In plants, physiological disorders refer to abnormalities or irregularities in the normal physiological processes that can affect their growth, development, and overall health. These disorders are often non-infectious and result from internal factors, environmental conditions, or a combination of both. Unlike diseases caused by pathogens, physiological disorders do not involve infectious agents such as bacteria, viruses, or fungi. Identifying the specific cause of a physiological disorder in plants is crucial for implementing appropriate corrective measures. Physiological disorders in onions and garlic can arise from various factors, including environmental conditions, cultural practices, and genetic factors. Here are some common physiological disorders that may affect these *Allium* crops: premature bolting, doubles, greening, sprouting, watery scale, thick neck, rubberization, premature sprouting, etc. (**Figure 1**). However, there is no single study that reviewed the progress of research in physiological disorders in onion and garlic.

Physiological disorders constitute a significant, yet often overlooked, economic threat to onion and garlic production. Studies report yield losses attributable to physiological disorders ranging from 10% to 50% in onion. The economic woes associated with physiological disorders extend beyond the field, as these disorders can increase susceptibility to spoilage during storage, leading to additional post-harvest losses. In onion, the losses due to physiological disorders vary along with the season and the varietal response/traits. In India onions are grown in three seasons viz; *Kharif:* planting in July-August, harvested in October-December. 2. Late *Kharif:* planted in October-November, harvested in January-February, *Rabi*: planted in December-January, harvested in March-May. In the *Kharif* season, the highest yield losses contributed by physiological disorders are due to post-harvest sprouting (54%) and double bulbs (up to 17%) (Annual report, ICAR-DOGR 2004; Annual report, ICAR-DOGR 2022). Whereas, in the late-*Kharif* season, the bolting (up to 23%) and double bulb (up to 25%) contribute more in losses (Annual report, ICAR-DOGR 2022). Comparatively, the post-monsoon (*Rabi*) onion production witnessed lesser yield losses due to physiological disorders.

Bibliometrics is a quantitative method used to analyze various aspects of scientific or academic literature (Ellegaard and Wallin, 2015; Wei et al., 2022, 2023). It involves the statistical analysis of publications, citations, and other bibliographic data to understand patterns, trends, and relationships within a particular field or body of literature. Further, this analysis is visualised using different graphical tools. In the present review, we conducted a bibliometric analysis of literature from 1946 to April 2024 on physiological disorders in onion and garlic retrieved from the SCOPUS database.

## 2 Bibliometric analysis

**Figure 2:**
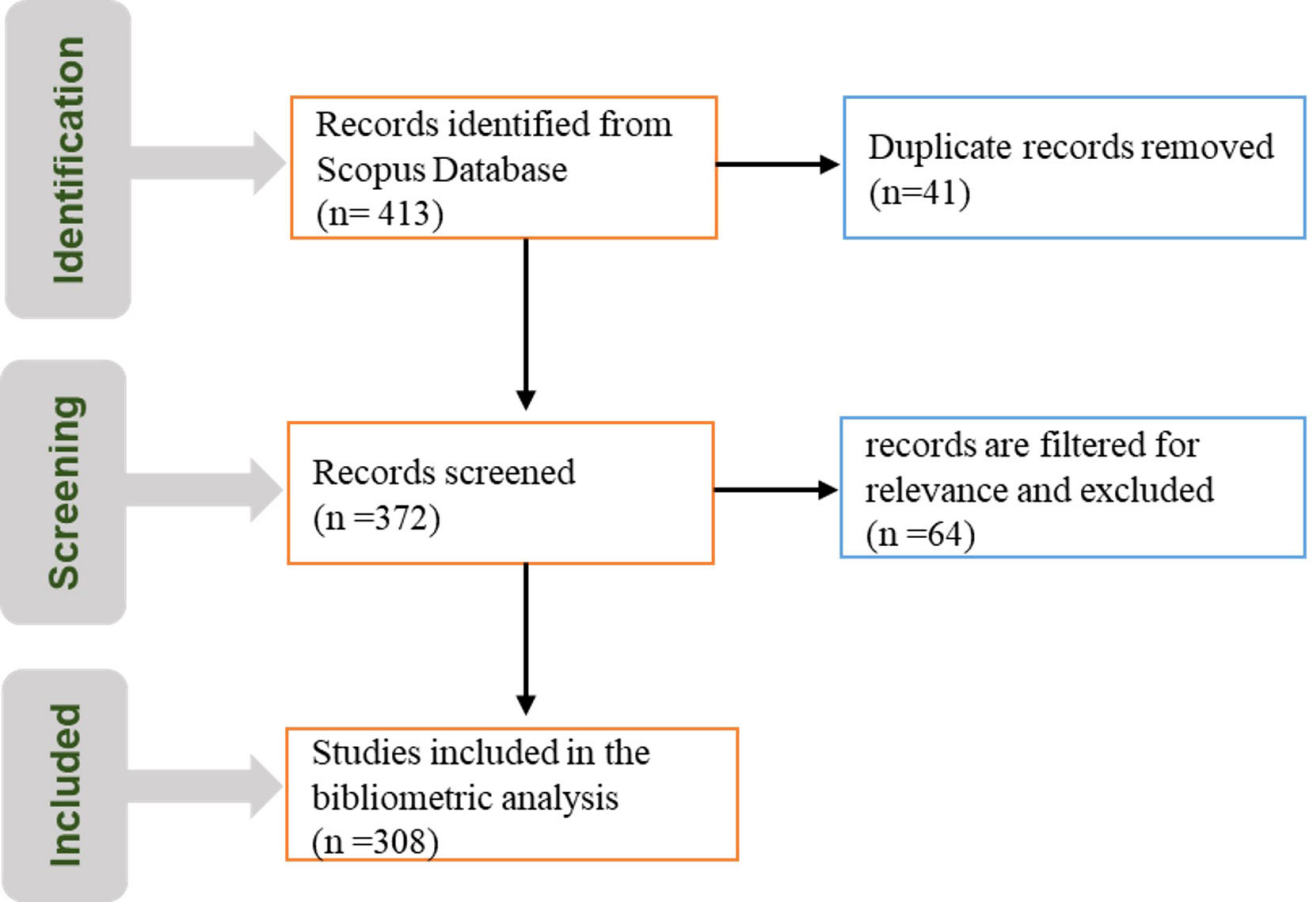
PRISMA diagram for bibliometric analysis on physiological disorders in onion and garlic.

Scopus is one of the most comprehensive scientific literature databases for bibliometric analysis (Baas et al., 2020). In the present analysis, we retrieved the relevant literature related to physiological disorders in onion and garlic from the Scopus database. The review of the literature was done using different keywords like ‘onion sprouting,’ ‘premature bolting,’ ‘watery scales,’ ‘premature sprouting,’ etc., using Boolean operators such as ‘AND’ and ‘OR.’ The searched terms should be present in the following fields: title, abstract, and keywords. A total of 308 documents published between 1946 to 2024 were retrieved. The data was exported from Scopus in CSV format for further bibliometric analysis. Then, these articles are analysed using Bibliometrix-Biblioshiny (Aria and Cuccurullo, 2017) and the VOSviewer version 1.6.20 (Van Eck and Waltman, 2010). These tools are widely used in bibliometric analysis and visualization due to their ease of use and clarity in representation. The detailed search strategy and data filtering process are shown in **Figure 2**.

The outcome bibliometric analysis is summarised in **Supplementary File 1**. The annual scientific production associated with physiological disorders in onion and garlic showed a steady increase over the last three decades, and most articles were published in the year 2016. However, the small number of articles published per year suggests the limited importance given by researchers to this area. India, the UK, and the USA are the top three countries with scientific production, but in the list of most cited countries, the UK is on the top, followed by the USA, Pakistan, and India, suggesting the importance of physiological disorders of onion and garlic in these countries. Though, China is one of the major producer of onion garlic, research output on physiological disorders is found limited. It might be due to that we restricted literature retrival to the English language only and some of the publications may not be present in the Socpus database. These major physiological disorders are largely influenced by environmental interactions. In South Asian countries, particularly India, multiseasonal onion cultivation contributes to a higher incidence of these disorders compared to China and other temperate regions, where onion cultivation is limited to a single season with long-day onion varieties. This may explain the increased research focus on these disorders in short-day onion regions.

**Figure 3:**
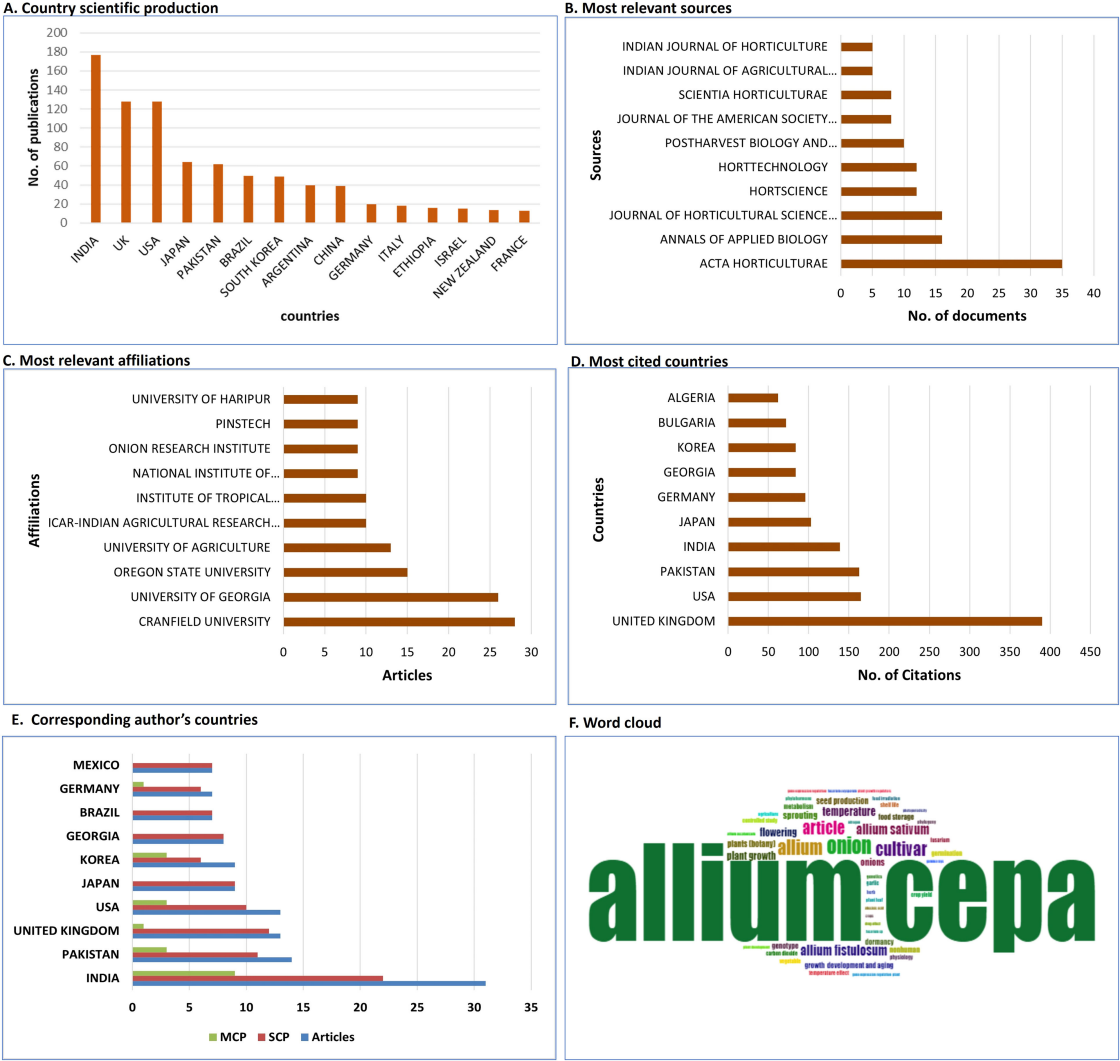
Analysis of different bibliometrics: **(A)** Country-wise scientific production, **(B)** Most relevant sources, **(C)** Most relevant affiliations, **(D)** Most cited countries, **(E)** Corresponding authors’ countries and **(F)** Word cloud map.

We further analysed the data for corresponding author countries and multiple and single-country publications. Both multiple-country publications (MCP) and single-country publications (SCP) are the highest in India. Whereas the USA, Pakistan, and Korea have almost similar MCPs. MCP tends to have an impact in terms of citations and collaborations, diverse expertise compared to SCP. Based on the data retrieved from Scopus, the most relevant source of scientific literature related to physiological disorders is found to be a journal named ‘Acta Horticulturae’ with the highest number of publications. This analysis showed that Cranfield University is the most relevant affiliation from which the highest number of research on physiological disorders of onion and garlic has been published. A word cloud generated from Biblioshiny provides an intuitive approach to the visualization of key themes in scientific literature to get insights into the research landscape of the field of interest. The size of a word is correlated to its frequency of occurrence. **Figure 3** depicts different bibliometric analyses.

### 2.1 Co-occurrence analysis of the author’s keyword

**Figure 4:**
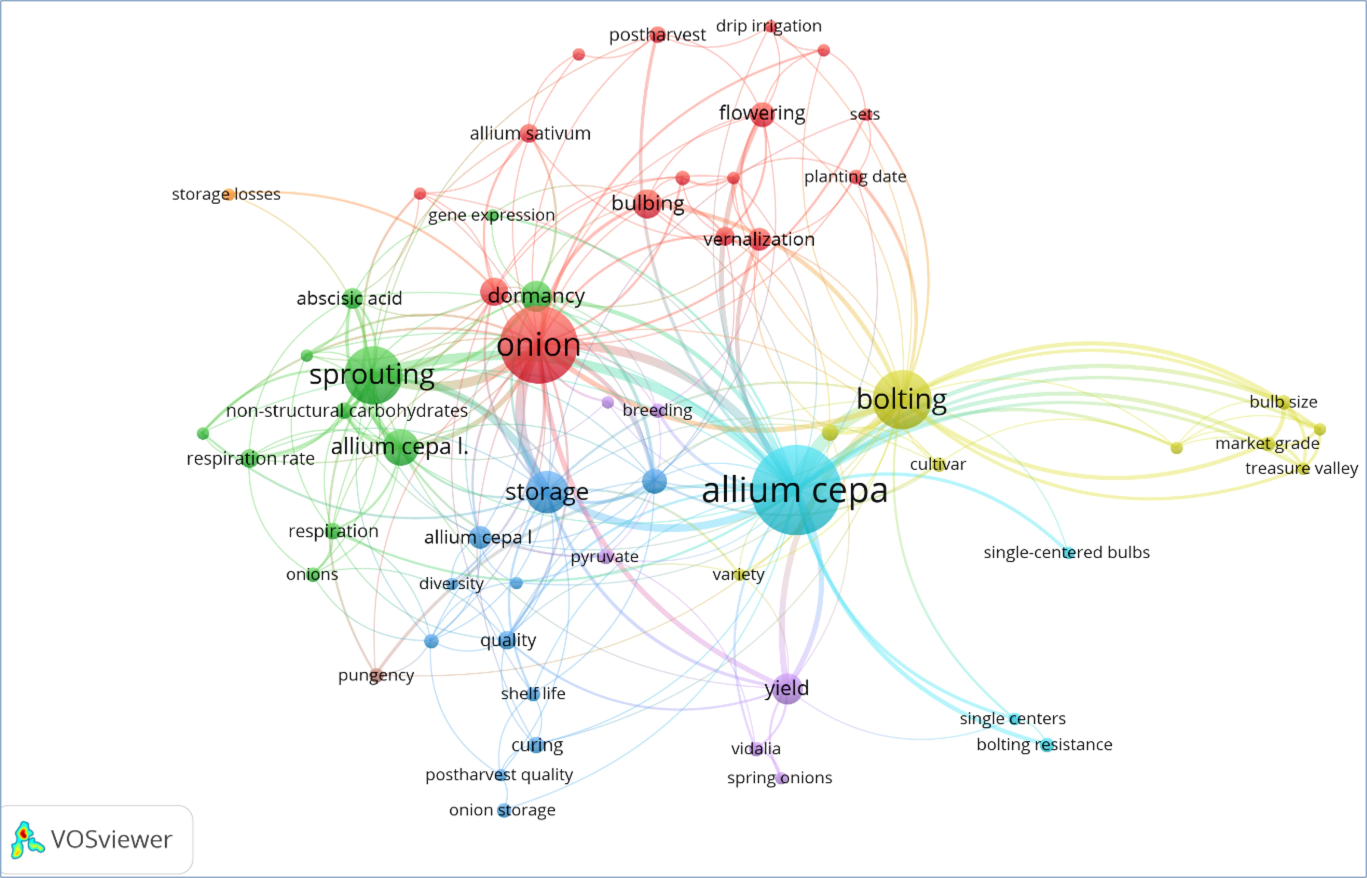
Co-occurrence analysis of author’s keyword in VOSviewer.

Keywords play a crucial role in the indexing of scientific literature databases as keywords suggest themes or aspects covered in particular research articles. A co-occurrence network of authors’ keywords was visualized using VOSviewer software (**Figure 4**). This analysis aimed to study the frequency and co-occurrence of these keywords found in the retrieved data. Out of a total of 677 author keywords, 59 met the criteria of a minimum three number of occurrences in the data. The present keyword co-occurrence network showed 8 clusters. The first four major clusters contained 47 keywords (Cluster 1 contained 16 items, Clusters 2 and 3 had 11 items, and Cluster 4 contained 9 items).

Bolting and sprouting are the major onion disorders, leading to significant economic losses. The present keyword co-occurrence network map depicted the two major clusters for these two disorders. **Figure 4** shows the network of keywords related to bolting, such as onion, variety, cultivar, vernalization, environment, planting date, sets, bulb size, storage, etc. Sprouting co-occurs with the following keywords: onion, garlic, respiration, storage losses, maleic hydrazide, abscisic acid, curing, dormancy, temperature, storage, control atmosphere storage, etc. Single-centred character is found to co-occur with cultivar, bolting, bulb size, market grade, and *Allium cepa*. From this bibliometric analysis, it can be concluded that most research is done on sprouting and bolting. However, the research community has not given much attention to other disorders. These disorders are reviewed in the subsequent sections.

## 3 Premature bolting

**Figure 5:**
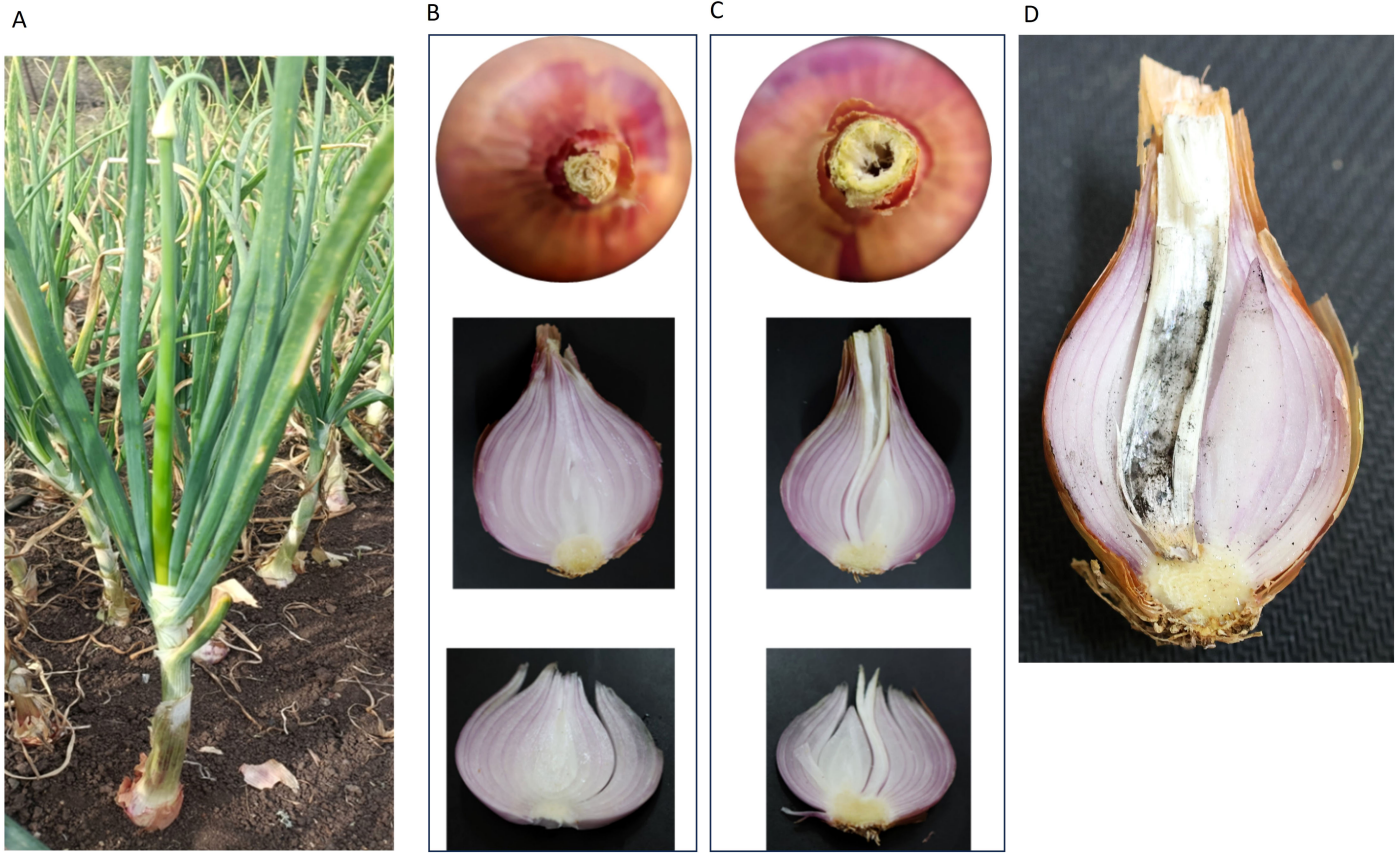
Undesirable bolting in onion crop, **(A)** Bolted onion plant in the field, **(B)** Healthy Non-bolter bulb, **(C)** Bolted bulb, showing dry spongy hollow space at the centre, **(D)** Fungal infection in the bolted bulb.

Bolting is premature flowering in the onion crop cultivated for bulb production. Premature flowering, i.e., seed stalks/scapes, are developed before the onion bulbs complete their normal life cycle (**Figure 5**). This transition in the plant life cycle is controlled by several endogenous and environmental signals such as temperature, photoperiod, nutrient management, varietal characteristics etc. It is an undesirable character as the further development of onion bulbs stops, and bulbs of such plants are light in weight, fibrous, and have a lower shelf life. It adversely affects the yield, quality, and, ultimately, income of the onion grower. The problem of premature floral stalk emergence poses a significant problem impacting the production and quality of onions. Estimates suggest potential damage of up to 30%, which varies with the climatic conditions and the planting date (Macías and y Grijalva, 2005). Due to the inferior quality of bolted bulbs, their storage life is shorter. In India, 5-80% bolting is reported in early *Rabi* and late *Kharif* seasons, which leads to an annual loss of approximately 11 to 50 hundred thousand tonnes of onion bulbs (Gorrepati et al., 2017).

It is well known that bolting negatively affects the yield and market value of onions. Researchers have studied these effects of bolting on onion in terms of TSS, sugar content, weight, pungency, phenolics content, storage life, etc. Kwon et al. (2016) studied the impact of premature bolting and its removal at various lengths on quality and growth. They observed that there was a reduction in weight without affecting the diameter of the bulb. Further, there was no change in the TSS, total sugar, pungency, and phenolic content. The author suggested that as biochemical properties are similar in both bolted and normal bulbs, they can be used in processing to prepare dehydrated powder, flakes, rings, etc. In contrast, Gorrepati et al. (2020) observed a significant difference in the TSS of bolted and non-bolted onions of four different varieties. However, the moisture and flavonoid levels did not vary significantly.

### 3.1 Factors involved in undesirable bolting in bulb onion

#### 3.1.1 Age of seedlings and planting time

Planting onions too early in the season may expose them to unfavourable conditions, leading to bolting. When plants were grown from large sets, more bolting was observed than those from smaller sets (Holdsworth 1945). Brewster and Salter (1980) observed that sowing in late August gave the highest bulb yield, whereas higher bolting was seen in early August sowings. Cramer (2003) demonstrated that later planting of bolting susceptible varieties can decrease the percentage of bolting. The study by Khokhar et al. (2007) demonstrated the significance of transplanting time and crop vegetative growth on the incidence of bolting events. They scientifically proved that low temperatures (6 to 15°C) and plants with a minimum of 7 to 10 leaves induce bolting in onion crops. The premature bolting in winter onion was found to be dependent on variety and sowing date in the Republic of Macedonia (Agic et al., 2007). Similarly, the difference in bolting percentage concerning the age of seedlings and planting date was also observed by Muhammad et al. (2021) in Pakistan. Bolting was found to increase with the increase in the age of seedlings transplanted, and the seedlings of the age of 45 days should be used to avoid bolting (Singh et al., 2012).

Verma et al. (2024) screened 122 onion varieties for their tendency to bolt prematurely across two seasons and revealed that early planting (November) led to more premature bolting compared to later planting (January). They characterized the genotypes based on phenotypic and molecular performance and identified the pre-mature bolting tolerance/resistance onion genotypes. Low temperature induces bolting events in onion crops however; it was observed that sensitivity to low temperature increases with an increase in plant size. The bolting process was stimulated when the crop was exposed to low temperatures at the growth stage of 7-10 leaves. This confirmed that early transplanting of onion seedlings during the late-*Kharif* season facilitates the crop to attain sufficient vegetative growth. And once the temperature falls particularly the night temperature, the crop stimulates pre-mature flowering instead bulbing process (Khan et al., 2021). Additionally, the study also evidenced that the incidence of bolting was higher during early *Rabi or late-Kharif* (0–76.67%) than during *Rabi* season (0–24.60%) directly reflecting the significance of Vernalization in the bolting process.

Furthermore, the previous reports (Khokhar et al., 2007) highlight the significance of transplanting time and crop vegetative growth on the incidence of bolting events. In an onion crop for bolting induction, a direct correlation was observed between the plant height and the number of leaves. The bolting process was stimulated when the crop was exposed to low temperatures at the growth stage of 7-10 leaves. This confirmed that early transplanting of onion seedlings during the late-*Kharif* season facilitates the crop to attain sufficient vegetative growth. Once the temperature falls particularly the night temperature, the crop stimulates pre-mature flowering instead bulbing process (Khan et al., 2021).

In Indian conditions, the majority of the onion variety planted was of short day and intermediate type where the bulbing process directly depends on day length and temperature. Previous findings evidenced the incidence of bolting that was higher during early *Rabi or* late*-Kharif* (0–77%) than *Rabi* season (0-25%) directly reflecting the significance of Vernalization in the bolting process. The previous study insights the bolting events in the popular onion growing region of Bengaluru and Bagalkot of southern India underlining the role of seasonality in stimulating the bolting tendencies of onion crop with the highest bolting rate of 15.09% during *Rabi* as compared to *Kharif* (0.21%) and summer (1.53%) seasons (Madalageri et al., 2024). Similarly, undesirable bolting in Welsh onion can be managed by proper selection of planting date and location (Dong et al., 2013).

#### 3.1.2 Varietal characteristics

Bolting in onion crops is not only determined by planting time and low-temperature exposure but also varies significantly with the cultivars. Some onion varieties are more prone to bolting than others. Choosing the right variety for the specific growing conditions is crucial. Holdsworth, in 1945, studied the onion varieties for their bolting characteristics when cultivated using sets and found diversity in bolting behaviour. A similar noticeable difference in susceptibility to bolting was observed by Brewster and Salter (1980). The yellow onion varieties NuMex BR1 and NuMex Sunlite were reported to be resistant to premature seed stalk emergence (Corgan, 1984, 1988). Gupta et al. (2018) screened onion germplasm for bolting tolerance during late *Kharif*. DOGR-1168 and DOGR-595 give the highest marketable yield with less than 5% bolting. Vandna et al. (2023) studied the behaviour of bolting in different varieties, and minimum bolting (2%) was reported in the Bhima Super variety. Verma et al. (2024) categorized 122 varieties based on their bolting resistance: resistant (less than 1% bolting), tolerant (1-10% bolting), susceptible (10-25% bolting), and highly susceptible (over 25% bolting). Interestingly, the varieties also showed a link between cold exposure and bolting rates. Gabriel et al. (2022), screened onion hybrids for premature flowering resistance and observed a flowering rate between 3 to 43%. A similar study in Welsh onion also demonstrated that an appropriate selection of variety could significantly control the bolting (Dong et al., 2013). This variation among the varieties might be due to the distinct genetic makeup of different varieties and their adaptability under diverse agro-climatic zones (Nayak et al., 2022; Hirave et al., 2015).

#### 3.1.3 Nutrient management

An imbalance in essential nutrients, particularly excessive nitrogen, can contribute to bolting in onions. Earlier, onions were designated as nitro-neutral plants; still, there are several reports describing the effect of N on flowering in onions (Brewster, 1983; Paterson, 1984). Research on the impact of nitrogen fertilizer on premature bolting in onions remains insufficiently explored. Díaz-Pérez et al. (2003) demonstrated that low N fertilizer led to premature bolting, whereas a steady increase in the N fertilizer application decreased the occurrence of bolting. According to Abdissa et al. (2011), the proportion of plants exhibiting bolting decreased significantly with applying N compared to the control. Similar findings were reported by Khan et al. (2019), and it was also found that bolting was not observed in onions, which are transplanted very late regardless of N nutrition. Low nitrogen levels were observed to promote bolting in bunching onions (*A. fistulosum* L.) (Yamasaki and Tanaka, 2005).

Nitrogen fertilizer alters the plant’s C/N ratio, and higher rates of nitrogen are likely to decrease the C/N ratio, reducing the incidence of bolting (Díaz-Pérez et al., 2003). It is crucial to apply the appropriate nitrogen fertilizer during the transition from the vegetative to the reproductive stage in onion plants. The second dose of nitrogen fertilizer should be administered just before the onset of bulbing to further lower the C/N ratio and prevent bolting (Khan et al., 2019). Likewise, bolting was promoted in bunching onions (*Allium fistulosum* L.) in response to low nitrogen levels (Yamasaki and Tanaka, 2005). The increased nitrogen level from 0 to 150 kg ha^-1^ reduces the bolting percentage by 62% (Gebretsadik, 2016). Thus, appropriate plant nutrition strategies involving mineral, organic and inorganic fertilizers or their combinations are vital for reducing the bolting incidence and increasing the bulb yield of onion crops.

#### 3.1.4 Temperature and photoperiod

In onion crops, flowering is mainly controlled by low temperature or vernalization and bulb formation by photoperiod. In India, short-day onion is grown in the plains and requires 10-12 hours day length. The long-day onion cultivated in hills requires 13-14 hours of day length. In short-day onion cultivars, bulb initiation takes place between 10-15°C night and 20- 25°C day temperature. Bulb development is best at 18-20°C night and 25-30°C day temperature. This indicates that for producing high-quality marketable bulbs, the crop requires low temperature during the initial growth phase followed by slightly higher temperature towards maturity (Rabinowitch and Currah, 2002). A shift in atmospheric temperature increases the incidence of bolting particularly in late*-Kharif* and *Rabi* crops. A sudden drop in temperature imparts a stress-like situation in plants that forces them to induce bolting as one of the survival mechanisms. This disturbs the normal photo-assimilate partitioning and develops the competition for resources in the simultaneously developing flower stalk and bulbs (Etoh and Simon, 2002). Most of the assimilation was diverted towards the young developing inflorescence that subsequently resulted in the development of small, lightweight, fibrous unmarketable bulbs with poor shelf life (Khan et al., 2019).

**Figure 6:**
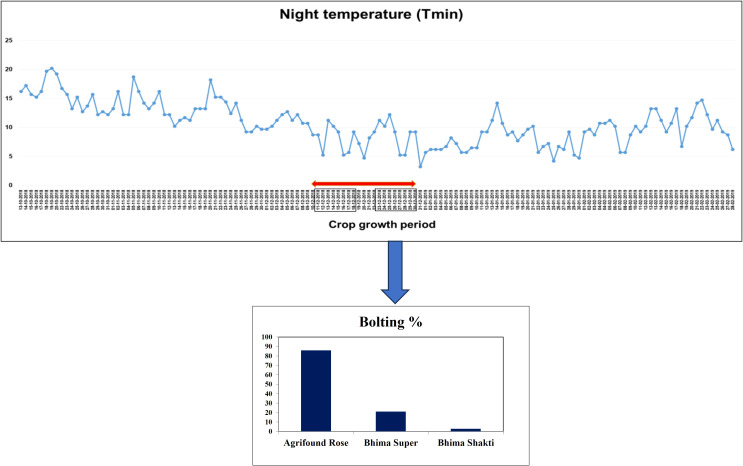
Effect of low night temperature of bolting of three different varieties.

The study conducted at ICAR-DOGR, Pune, highlights the bolting behaviour in the popular onion variety. The data suggests that low temperatures (less than 10°C) for more than one week during November-December induce bolting in late-*Kharif* crops as shown in **Figure 6**. Onions are sensitive to day length, and certain varieties may bolt if exposed to longer days than they require. Onions are sensitive to changes in daylight duration. Exposure to conditions outside their optimal photoperiod can trigger premature bolting. Khokhar et al. (2007) demonstrated that the cold storage of sets leads to more bolting than sets stored at 20°C. Further, bolting was found to be increased with the set size and the low-temperature storage (Khokhar, 2009).

#### 3.1.5 Growth regulators

Growth regulators and phytohormones play vital roles in the growth and development of plants. These growth regulators, especially growth retardants, can be used in preventing undesirable bolting in onions and other crops. These growth retardants inhibit the synthesis of gibberellin, thereby decreasing vegetative growth (e.g., Maleic hydrazide). Some growth regulators led to the release of ethylene inside the plant cell, which accelerates the process of maturity and inhibits bolting (e.g., ethephon). Choudhri and Bhatnagar (1954) used the Maleic hydrazide treatments to avoid premature bolting and subsequently increase onion yield and quality. Ethephon (5000 ppm) has been employed to diminish premature bolting, and earlier studies suggest a reduction of the issue by as much as 22% (Cantliffe and Woods, 1978; Corgan and Izquierdo, 1979). Recently, Macías Duarte et al. (2017) evaluated the effect of different growth regulators on the premature emission of the floral stalks. A significantly reduced bolting (10.5%) was observed in the plot treated with the ethephon (5000 ppm) and Mepiquat Chloride (42 ppm) compared to the control (30.7%). ‘Paclobutrazol’ a triazole derivate is well known for its ‘Anti-Gibberrellic property. It regulates the isoprenoid pathway, inhibiting GA biosynthesis, declining ethylene production and enhancing the abscisic acid (ABA) and cytokinin biosynthesis (Bista et al., 2022). In this context, the use of Paclobutrazol @ 20 to 40 ppm reduces bolting and enhances the onion bulb yield by increasing the bulb size (Arvin and Banakar, 2002; Ashrafuzzaman et al., 2009).

### 3.2 Genetic/molecular studies

Controlling the timing of bolting in onions is a crucial strategy for enhancing bulb and seed production. Nevertheless, there are only a few genetic and molecular studies addressing bolting in onion. Genetic and molecular studies must be undertaken to elucidate the mechanisms governing bolting time to avoid the losses of yield and quality of onion. Hyun et al. (2009) first time attempted the genetic analysis of bolting in onion and, after crossing between early and late bolting genotypes, concluded that late bolting is governed by a dominant locus. Further proteomics analysis indicated that there might be a link between histone modification/chromatin remodelling and bolting in onions. Baldwin et al. (2014) performed the first linkage mapping study of candidate genes implicated in photoperiod and vernalization physiology and QTL for premature bolting in onion. A selective genotyping of the F_2_ family derived from ‘Nasik Red × CUDH2150’ revealed that specific regions on chromosomes 1, 3, and 6 were found to be associated with bolting. A QTL consistently conditions bolting susceptibility in this cross on chromosome 1, which was named *AcBlt1.* Another study by Lee et al. (2013) reported that the AcFT2 gene is involved in the induction of bolting in response to vernalization.

Verma et al. (2024) showed the genetic difference between bolting susceptible and tolerant onion varieties using 36 SSR markers. This study also revealed varieties needing a longer cold period were less likely to bolt prematurely. This finding highlights the connection between genes and bolting behaviour at both the morphological and genetic levels. Late bolting is an important agronomic trait in bunching onion (*A. fistulosum* L.) due to its profound impact on yield and quality. Wako et al. (2016) first time shed light on the genetic underpinnings of bolting time in bunching onion in two F2:3 populations resulting from the crossing of early and late bolting genotypes. QTL analysis revealed a single major QTL situated exclusively on the linkage group Chr. 2a, which is closely linked to the same SSR marker in both the population studied.

## 4 Bulbil formation

**Figure 7:**
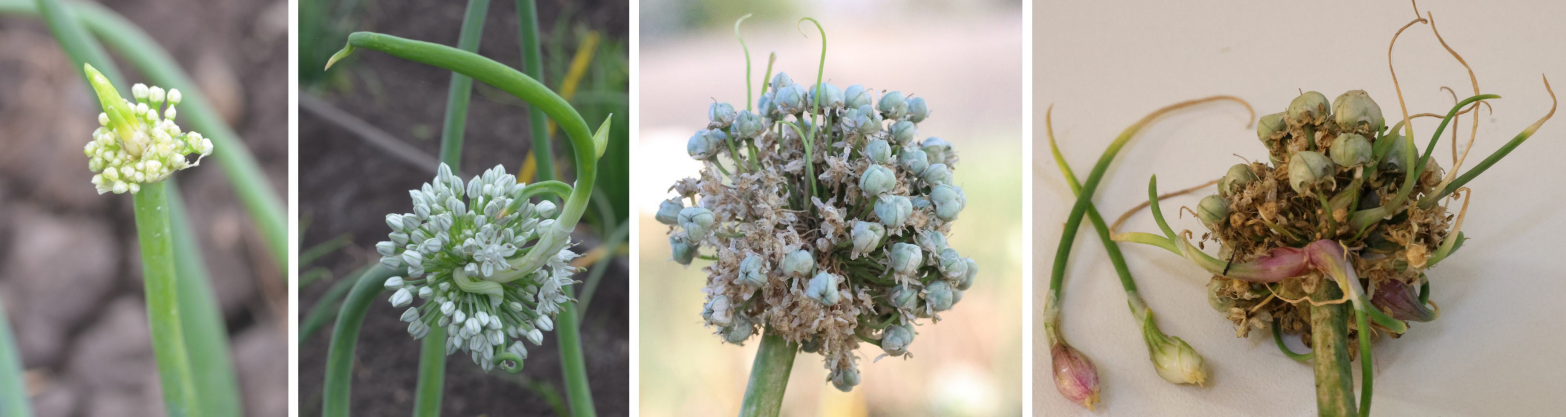
Different types of deformities in onion flower.

Due to environmental factors, bulbil formation in flowers instead of seed development in onions is observed in certain genotypes. Though it is a natural phenomenon in *Allium* x *proliferum* (also called as Egyptian onion, tree onion, walking onions), these bulbils can be utilized for the propagation of vegetatively proppagted onion (Kamenetsky and Rabinowitch, 2006). However, it is considered undesirable in onion seed production plots as it reduces the yield of the seeds, which have to compete with these bulblets or topsets. We observed this anomaly in a few onion flowers in seed production plots where bulbils are observed instead of seed development (**Figure 7**). Hasanov and Akparov (2018) observed that hot weather conditions resulted in this disorder in few flowers. Propagation with these bulbils or topsets has some disadvantages, such as lower yield and difficulty in early establishment crops, i.e., more mortality.

## 5 Sprouting

**Figure 8:**
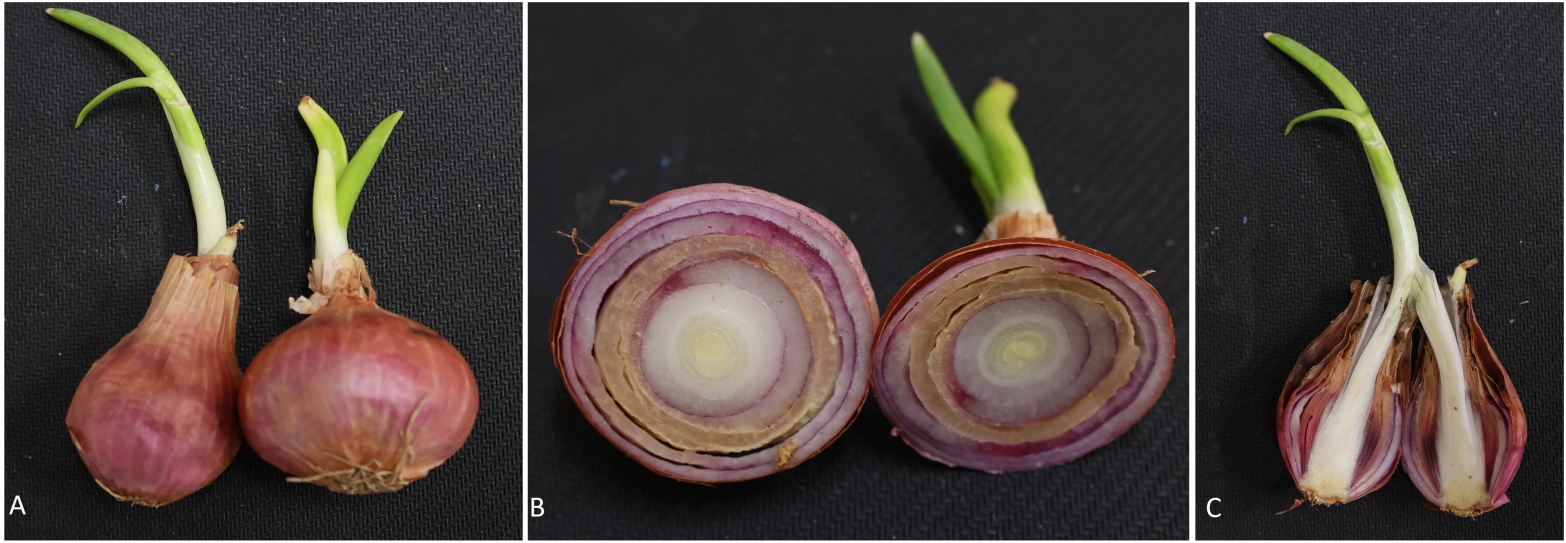
Onion sprouting **(A)** Sprouted onion bulbs, **(B)** Cros section and **(C)** Vertical section showing sprouting associated bulb deterioration.

Sprouting can alter the flavour and texture of the onion, making it less desirable for the market. In storage, significant loss occurs due to the dormancy break and eventual sprouting of onions (Adamicki, 2005). Onions are harvested after the completion of the bulbing phase, i.e., after neck fall and senescence of foliage. At this stage, bulbs are in endo-dormancy. After some time in storage, the bulbs start to break dormancy, and the emergence of sprouts can be observed (**Figure 8**). This dormancy period varies with genotype. This dormancy period can be extended by cold temperature storage and the use of growth regulators (Forney et al., 2022). Several factors are involved in the sprouting of onions such as genotype, soil moisture at the time of harvest, fertilizer management, physiological maturity at harvest, curing, etc. (Miedema, 1994; Sorensen and Grevsen, 2001; 2004; Gorrepati et al., 2017). Geisseler et al. (2022) reviewed the role and requirement of nitrogen fertilizer in onion crop development and concluded that excess N fertilizer led to higher sprouting and bulb rotting.

The exact period when dormancy breaks in onion is still not completely understood. During dormancy break or sprouting, several biochemical and molecular changes occur in the bulb, such as growth regulator balance, modification of cell wall, carbohydrate mobilization, fructan redistribution, etc. Ohanenye et al. (2019) reported that fructan redistribution could be considered a marker for predicting onion bulb dormancy break. Recently few studies investigated the sprouting in onion at transcriptome and transcription factor level. Another study by Kleman et al. (2024) revealed that regardless of maleic hydrazide use the levels of fructose and glucose were linked to the degree of sprouting, with the highest levels occurring at the onset of sprouting in spring. Additionally, dry matter content was significantly correlated with the proportion of sprouted bulbs in a sample. Alamar et al. (2020) concluded that ethylene modulates the expression of several genes, such as ACO, EIN4, and EIL3 to extend the dormancy. Puccio et al. (2022) demonstrated the key role of *AcWRKY32* in onion bulb dormancy release. These studies are encouraging the researchers to extend the dormancy of onion to control the losses due to sprouting.

### 5.1 Control measures to avoid sprouting

Harvesting at complete maturity, proper curing, use of growth regulators, and irradiation are a few measures to control or minimize the undesirable sprouting in the onion. The sprouts and rooting of onion bulbs were completely inhibited after a preharvest spray of 2500 ppm maleic hydrazide up to 5 months of storage at 55°F (Wittwer et al., 1950). The maleic hydrazide disrupts the onion meristem and disturbs the cell division, thereby reducing the sprouting (Isenberg et al., 1974). Although maleic hydrazide (MH) effectively controls sprouting in onion storage its use was completely excluded as it causes chromosomal aberrations in animals and mammalian cells (Marcano et al., 2004). Due to the banning of MH by the Government of India in 2009, there is a need of an hour to identify the alternative option that can resemble the effect produced by MH.

Chope et al. (2007a), for the first time, demonstrated that the use of 1-methylcyclopropene significantly delayed sprout emergence. The use of ethylene or 1-methylcyclopropene was reported to show a reduction in the sprout growth compared to the control, especially after the curing (Bufler, 2009; Cools et al., 2011; Forney et al., 2022). The curing is one of the important factors which determine the post-harvest storage of onion. Abscisic acid (ABA) is a key enzyme that determines the dormancy of the onion bulbs; there is a rapid decrease in the ABA level between harvest and initial days of storage, and it might be due to the curing at high temperatures (Chope et al., 2007b). Properly curing onion can reduce post-harvest losses due to sprouting, rotting, and physiological weight loss (Gorrepati et al., 2017; Nega et al., 2015). The irradiation of onion bulbs is widely accepted and is used to inhibit unwanted sprouting in onion. Irradiation inhibits sprouting and reduces the losses due to weight loss and rotting (Farooqi and Donini, 1976; Sharma et al., 2020). Kavita et al. (2024) observed an increase in flavour volatiles, sulfur compounds, quercetin, and antioxidant capacity of gamma-irradiated onion bulbs.

## 6 Watery/leathery scale in storage

Watery scale is an economically important physiological disorder observed in stored onion. The onion exhibits sensitivity to elevated carbon dioxide levels, whether found within its internal scales or in the external atmosphere during storage in controlled atmosphere (CA) storage facilities. This higher CO_2_ level led to the development of a physiological disorder called watery scale. Adamicki et al. (1977) found that CO_2_ of 10% in CA storage resulted in the incidence of watery scales. Further, the same disorder was reported in all bulbs stored in polythene bags containing 10% CO_2_. Microscopic studies revealed the damage to the cell walls, which might be due to the activity of hydrolytic enzymes. The content of free amino acids was also found on the higher side in bulbs having this disorder than in normal bulbs. Therefore, proper storage condition needs to be provided to increase the shelf life of onions in storage. Hoftun (1993) demonstrated that the majority of gas exchange in onion bulbs occurs through the neck, and the watery scales incidence was noted when the CO_2_ levels exceeded 13% and oxygen (O_2_) levels fell below 4% in the internal atmosphere. The observation suggests that onions exhibit a higher sensitivity to elevated CO_2_ levels than to reduced O_2_ levels.

The symptoms and causes of watery scale disorder were described in detail by Solberg (2015) based on visual observation and biochemical analysis; he grouped the symptoms as follows: leathery scale: thick, dark scales between outer dry and inner fleshy scales; translucent scale: glassy, firm fleshy scales. Watery scale disorder is found to be linked to disruptions in the gaseous exchange of bulbs, which are characterized by elevated internal CO_2_ and/or reduced internal O_2_ levels. Higher humidity is also reported to be correlated with the watery scale of onion (Solberg Øivind and B⊘e, 1999). Further, it is also noted that watery scale is also a one of the symtoms of bacterial soft rot caused by bacterial pathogen *Pectobacterium carotovorum* subsp. *Carotovorum*. Similarly, injury also may cause the translucent scale, thus it is a complex disorder (Shock et al., 2003).

## 7 Splits/doubles

**Figure 9:**
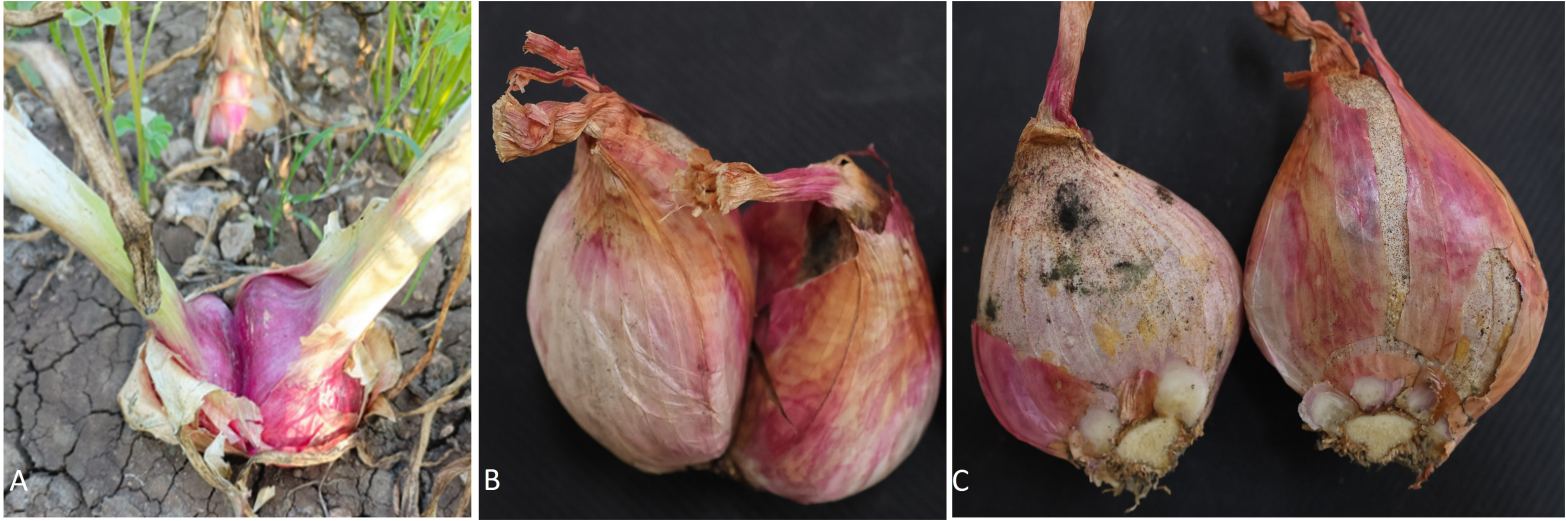
Doubles/splitting of onion bulbs, **(A)** Doubled onion in the field, **(B)** Doubled onion after harvesting, **(C)** Doubled onion bulb separated showing its attachment at the basal plate.

Bulb splitting in onions, also known as bulb doubling or twinning, refers to a phenomenon where a single onion bulb divides into two or more separate bulbs. This occurrence can result in multiple bulbs growing from a single root, each developing into a distinct onion (**Figure 9**). While some factors, such as specific environmental conditions or nutrient imbalances, may contribute to bulb splitting, it is generally considered an undesirable trait in commercial onion cultivation. Growers typically aim for single, well-formed bulbs, which are more marketable and easier to manage.

The incidence of this disorder is reported to be influenced by the genetics of the plant, climatic conditions, nutrient management, depth of planting, spacing between the plants, etc (Hassan, 1984; Thompson, 1934; Rabinowitch, 1979). The higher doubling was observed in plants developing from the larger sets than smaller ones, irrespective of short-day or long-day cultivars (Thompson, 1934; Rabinowitch, 1979). The application of higher nitrogenous fertilizer late in the growing season may accelerate bulb splitting (Brewster and Butler 1989). In 1984, Hassan noted that higher nitrogen application correlated with an increase in bulb doubling in onions, whereas potassium application resulted in the least occurrence of double bulbs. The higher nitrogenous fertiliser doses increased the count of double bulbs (Syed et al., 2000). Mechanical injury to developing bulbs during intercultural operations may lead to the emergence of outgrowth, and it might be one of the reasons for bulb splitting and doubling in onion.

## 8 Basal plate split

This type of bulb deformity is found worldwide, and its symptoms are: the initial indication noticed is the division of the basal plate, and then several small bulbs emerge from the divided basal plate of the affected bulb (https://www.vegetables.bayer.com/za/en-za/resources/disease-guides/onions/bulb-splitting.html). Such bulbs are not marketable and lead to economic losses for growers. Irregular irrigation practices increase the occurrence of this type of bulb deformity. The repeated over-irrigation followed by a dry spell increases the percentage of split bulbs. Such splitting of the basal plate provides passage for entry to another pathogenic organism, which further deteriorates the bulbs, aggravating this disorder. Proper land preparation and regular and precise water and nutrient management can help in reducing the incidence of this disorder.

## 9 Internal doubles/multi-centred bulbs

**Figure 10:**
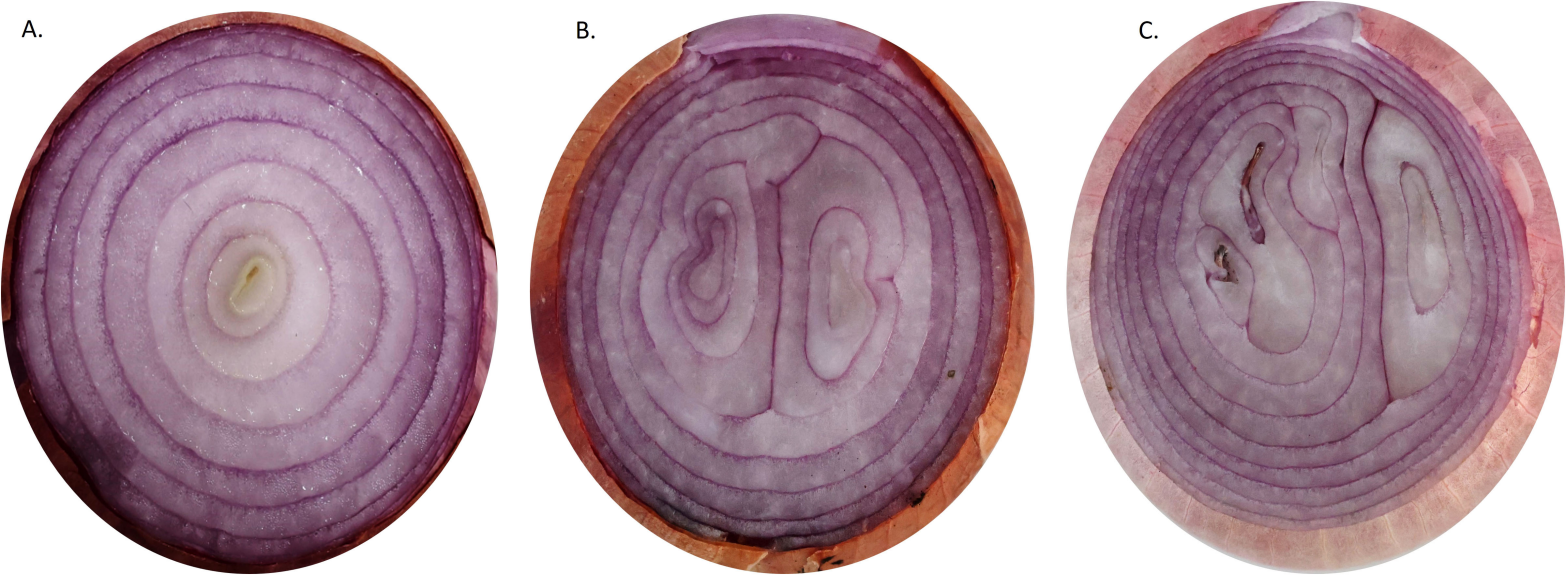
Transverse section of onion bulbs showing single **(A)** and multiple centres **(B, C)**.

A “single-centred onion” usually refers to an onion bulb that has only one central growth point from which the layers of the onion develop (**Figure 10**). Though this is not a physiological disorder in general, it can affect the use of onions for specific purposes. The single-centred onion bulbs are preferred by onion ring industries and restaurants for fresh use. Several factors affect the formation of a single-centred onion, such as genetics, nutrition, water management, stress, etc. The heritability of single-centred traits in different populations of short-day and intermediate-day type onion was studied and suggested that improvement can be made through selection (Wall et al., 1996; Cramer, 2006). This trait is found to be varied with the cultivars, and three years of evaluations of yellow cultivars showed that the single centre bulbs were in the range of 1 to 74% (Shock et al., 2005). Water stress at early growth stages (3 and 7, 3, 5-leaf stages) significantly reduced the single-centered bulbs (40%, 32%, and 18%, respectively) (Pelter et al., 2004). Similarly, Shock et al. (2007) also reported that onions proved susceptible to developing multiple centres under water stress, particularly during the 4 to 6-leaf stages of growth. Still, the physiological and molecular mechanism behind the single-centeredness in onion is not completely understood.

## 10 Thick neck

The thick neck in an onion occurs when the bulbs fail to mature, and there is continuous production of leaves; this leads to the thickening of the neck (pseudostem). It is also called incomplete bulbing (Brewster et al., 1987). It was observed that the application of nitrogenous fertilizer late in the season increases the pseudostem diameter and eventually produces thick-necked bulbs (Brewster and Butler 1989). The thick-necked bulbs deteriorate in storage rapidly due to sprouting and rotting. Previous literature searches revealed the correlation between thick neck and N application Tekeste et al., 2018; Khan et al., 2019). Some farmers are completely avoiding the use of urea to avoid the development of neck thickness and doubles (Palaniappan and Thangasamy, 2015). However, in addition to higher N application, neck thickness is also influenced by variety, location, transplant size, and season of planting (Brewster et al., 1987; Mettananda and Fordham, 1999).

## 11 Premature sprouting and splitting in garlic

**Figure 11:**
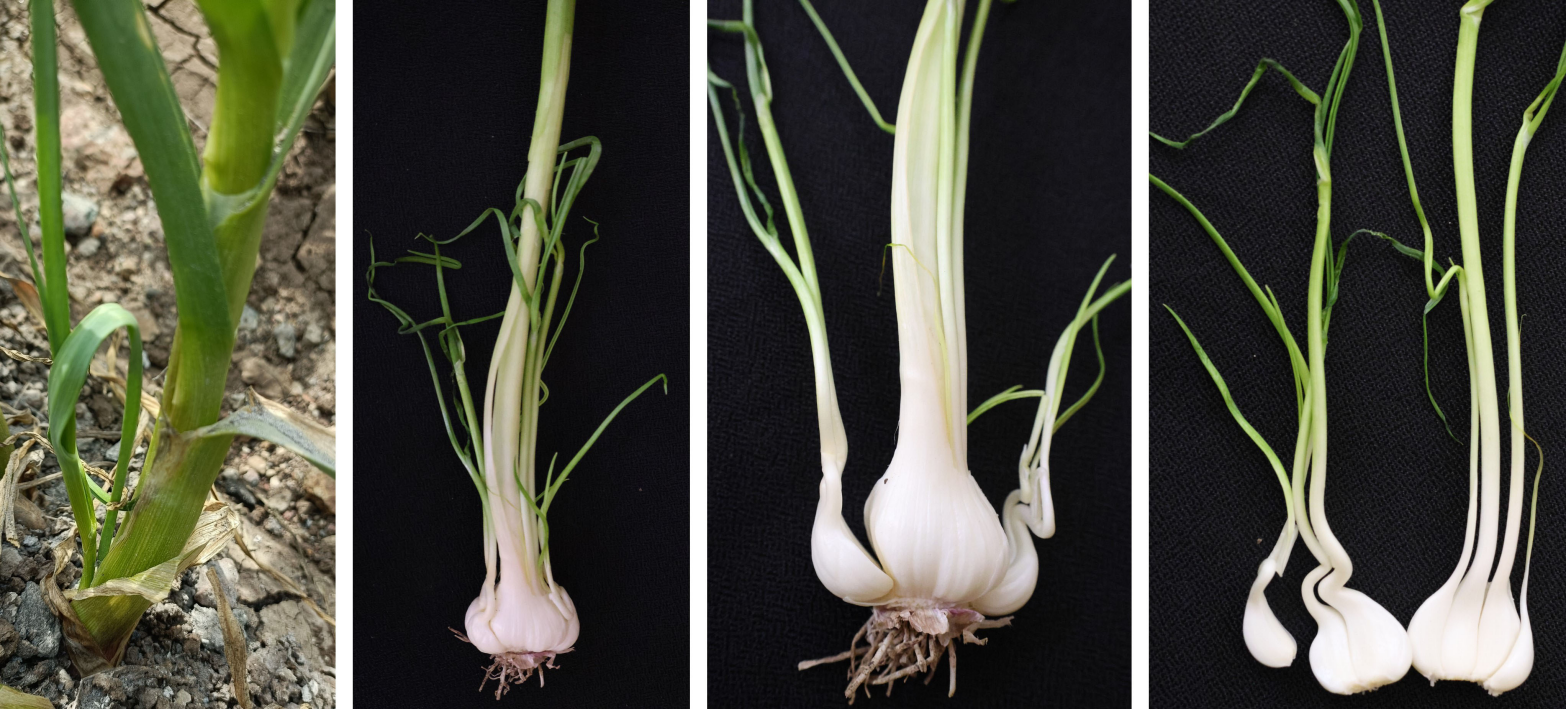
Premature sprouting in garlic.

Premature sprouting is a physiological anomaly wherein cloves, post-initiation, persistently develop shoots in the field, generating leaves instead of forming bulbs. These sprouts emerge through the leaf sheath, causing cloves to split and diminishing the market quality of the bulbs (**Figure 11**). The varietal difference in premature sprouting of garlic bulbs in the field before harvest was studied, and found that white varieties are more susceptible to sprouting than purple varieties. Further, higher plant density resulted in less tendency towards sprouting (Garcia, 1980). Islam et al. (2015) evaluated garlic germplasm for premature sprouting under mulched conditions at multiple locations and found that premature sprouting ranged from 0.07% to 42%. Another study of screening for premature sprouting of garlic in Bangladesh reported 0.4% to 42% sprouting (Sarker et al., 2017). Such a varietal difference in the sprouting of garlic indicates a wide genetic variation for this trait. Generally, excessive soil moisture and more nitrogen are key factors responsible for the premature sprouting of garlic in the field. Further, delays in harvesting and irrigation after a longer dry period sometimes led to the splitting of garlic bulbs.

## 12 Rubberization of garlic

Rubberization is one of the physiological disorders in garlic bulbs. It is called rubberization/rubberification because the clove becomes elastic as a rubber. When you apply pressure with your finger, it leads to dent formation, and after the release of pressure, it comes to normal. These affected bulbs acquire a spongy texture after drying (Satyagopal et al., 2014). A correlation study of different factors influencing rubberization in garlic demonstrated that thrips infestation and higher doses of nitrogen were the main causes of rubberization (Selvaraj et al., 1994).

### 12.1 Reasons for physiological disorders in garlic

As per AESA based IPM package for garlic (Satyagopal et al., 2014); sprouting and rubberization are mostly observed in low-lying areas with higher field deposition of nutrients, frequent irrigated fields, higher N fertilizer application, especially in the form of urea, higher moisture at maturity, delayed harvesting, widely spaced planting of cloves, etc.

## 13 Conclusion and future perspective

The physiological disorders in onion and garlic deteriorate the quality of produce. Therefore, these various disorders need to be managed with the help of adopting good agricultural practices, selecting suitable varieties, planting proper seasons, and following recommended post-harvest management. The detail recommendation for control of these disorders are mentioned in **Table 1**. Therefore, agriculture extension workers and farmers need to know the reason for, symptoms of, and prevention of these different physiological disorders occurring in Alliums. Bibliometric analysis revealed that among these disorders, bolting and sprouting were more given attention by researchers while other disorders seem to be neglected. Thus, the present review emphasizes that physiological disorders must be given equal importance to understand the physiological and molecular factors behind them and devise a proper cultivation practice to reduce the economic losses of growers.

**Table 1:** Reccomondation to control physiological disorders in onion and garlic.

Disorders	Recommendations to avoid these disorders
Bolting	Timely transplanting of healthy seedlings (45-50 days old seedlings) Use of bolting tolerant recommended onion varieties Balanced and recommended dose of fertilizers to maintain C/N ratio Use of growth regulators to avoid bolting (Ethylene spray)
Sprouting	Avoid excess use of nitrogen fertilizer Harvesting at complete physiological maturity Proper curing and grading Use of growth regulators to avoid sprouting (1-MCP and Ethylene) Maintain optimum humidity and temperature in storage structure Irradiating with gamma rays inhibits sprouting and rotting in stored onion
Watery scale	Ventilate storage areas to maintain optimum carbon-dioxide (below 10%) and oxygen (above 4%) level in controlled atmosphere storage Maintain optimum humidity and temperature in storage structure Avoid sealed packaging with high carbon-dioxide accumulation, prevent physical injury to bulbs
Split and Doubles	Avoid late-season applications of high nitrogen fertilizers and ensure sufficient potassium fertilization Use of small to medium size sets reduce doubles Planting with sufficient spacing and at optimum depth Avoid physical damage during various cultural operations
Internal doubles/multi-centred bulbs	Use cultivars that yield more single-centered bulbs Conduct multi-year selection and breeding for tendency toward single-centeredness Follow regular irrigation schedule and avoid early-stage water stress Follow recommended fertilizer dose to provide balanced nutrition for getting uniform bulb size
Premature sprouting in garlic	Use of recommended tolerant varieties Maintain higher plant density Avoid excess irrigation and nitrogen fertilizer at later growth stages Harvest at proper physiological maturity stage
